# An Elevated Glycemic Gap is Associated with Adverse Outcomes in Diabetic Patients with Acute Myocardial Infarction

**DOI:** 10.1038/srep27770

**Published:** 2016-06-13

**Authors:** Wen-I Liao, Chin-Sheng Lin, Chien-Hsing Lee, Ya-Chieh Wu, Wei-Chou Chang, Chin-Wang Hsu, Jen-Chun Wang, Shih-Hung Tsai

**Affiliations:** 1 Department of Emergency Medicine, Tri-Service General Hospital, National Defense Medical Center, Taipei, Taiwan; 2 Division of Cardiology, Department of Internal Medicine, Tri-Service General Hospital, National Defense Medical Center, Taipei, Taiwan; 3 Division of Endocrinology and Metabolism, Department of Internal Medicine, Tri-Service General Hospital, National Defense Medical Center, Taipei, Taiwan; 4 Graduate Institute of Aerospace and Undersea Medicine, National Defense Medical Center, Taipei, Taiwan; 5 Department of Radiology, Tri-Service General Hospital, National Defense Medical Center, Taipei, Taiwan; 6 Department of Emergency Medicine, School of Medicine, College of Medicine, Taipei Medical University, Taipei, Taiwan; 7 Department of Emergency and Critical Medicine, Wan Fang Hospital, Taipei Medical University, Taipei, Taiwan

## Abstract

Acute hyperglycemia is a frequent finding in patients presenting to the emergency department (ED) with acute myocardial infarction (AMI). The prognostic role of hyperglycemia in diabetic patients with AMI remains controversial. We retrospectively reviewed patients’ medical records to obtain demographic data, clinical presentation, major adverse cardiac events (MACEs), several clinical scores and laboratory data, including the plasma glucose level at initial presentation and HbA1c levels. The glycemic gap, which represents changes in serum glucose levels during the index event, was calculated from the glucose level upon ED admission minus the HbA1c-derived average glucose (ADAG). We enrolled 331 patients after the review of medical records. An elevated glycemic gap between admission serum glucose levels and ADAG were associated with an increased risk of mortality in patients. The glycemic gap showed superior discriminative power regarding the development of MACEs when compared with the admission glucose level. The calculation of the glycemic gap may increase the discriminative powers of established clinical scoring systems in diabetic patients presenting to the ED with AMI. In conclusion, the glycemic gap could be used as an adjunct parameter to assess the severity and prognosis of diabetic patients presenting with AMI. However, the usefulness of the glycemic gap should be further explored in prospective longitudinal studies.

Acute hyperglycemia is a frequent finding in patients presenting to the emergency department (ED) with acute myocardial infarction (AMI). The admission blood glucose level after AMI is an independent predictor of long-term mortality in patients with or without diagnosed diabetes^1^,^2^. An elevated blood glucose level may reflect a stress response and/or an underlying abnormal glucometabolic state. Regardless of the mechanism, an AMI complicated by hyperglycemia is associated with an inflammatory and prothrombotic state, depressed myocardial contractility and increased short- and long-term mortality^3^,^4^.

Stress-induced hyperglycemia (SIH) commonly occurs in patients with critical illnesses, such as sepsis, multiple trauma, burn injuries, major surgeries, and AMI^5^. Nonetheless, there are discordant findings on the correlation between hyperglycemia and adverse outcomes in acutely ill patients with or without preexisting diabetes^6^,^7^,^8^.

Because hyperglycemia is the cardinal feature of diabetes, it is necessary to consider pre-existing hyperglycemia in diabetic patients when investigating the association between SIH and adverse outcomes. There is a well-known correlation between glycated hemoglobin (HbA1c) and the long-term mean plasma glucose levels from the preceding 3 months. As estimated long-term average glucose level can be calculated from the HbA1c value with the equation derived from an international multicenter study of HbA1c-derived average glucose (ADAG)^9^. Therefore, we aimed to identify the major contributor to acute serum glucose levels. In acutely ill diabetic patients, the epiphenomenon of admission hyperglycemia may be caused by acute physiological stress and/or higher chronic baseline blood glucose levels^10^.

Although the role of hyperglycemia has been well studied as an index for poor prognosis in non-diabetic patients with AMI, the prognostic role of hyperglycemia in diabetic patients remains controversial. We hypothesized that an elevated glycemic gap is associated with an increased risk of developing major adverse cardiac events (MACEs) in diabetic patients presenting to the ED with AMI. The aim of the present study was to explore the correlation between the glycemic gap and adverse clinical outcomes; in addition, we sought to justify the use of the glycemic gap as a biomarker for the severity of diabetic patients presenting to the ED with AMI.

## Materials and Methods

### Patients

We conducted a retrospective observational study of consecutive patients with diabetes mellitus (DM) admitted for AMI between June 1, 2011 and June 31, 2015. The institutional review board for human investigations of a tertiary referral medical center at Tri-Service General Hospital approved this study and waived the necessity for informed consent. The methods were carried out in accordance with approved guidelines. The identification of patients with DM and AMI was performed by searching for the International Classification of Diseases (9th revision) codes 410 and 250.2–8. Diabetes was considered to be present if a patient was discharged from a hospital with a diagnosis of type 1 or type 2 diabetes, at least 1 prescription for insulin or an oral antidiabetic agent, and/or an HbA1c level ≥6.5% during the preceding 2 months^11^,^12^. In accordance with the global taskforce recommendation, AMI was diagnosed based on clinical and laboratory evidence of myocardial necrosis and ischemia^13^. The records of identified patients were reviewed manually to select AMI patients with data for plasma glucose levels at initial presentation and HbA1c levels obtained between 1 month prior to or immediately after their admission. We excluded patients with missing HbA1c or initial serum glucose levels, those on steroid treatments or presenting with hypoglycemia (blood glucose <70 mg/dL) and patients who received a blood transfusion.

### Methods

We retrospectively reviewed patients’ medical records to determine age, sex, underlying comorbidities, clinical presentation, and laboratory data, including B-type natriuretic peptide (BNP) and plasma glucose levels at initial ED presentation, HbA1c levels, and lengths of stay in the intensive care unit (ICU) and hospital. In-hospital MACEs, including AMI, emergency revascularization, cardiogenic shock or death (unless established as non-cardiac in origin), were used as the primary end point. We quantified the severity of the index AMI events using Global Registry of Acute Coronary Events (GRACE) scores and the Killip classification. GRACE scores were calculated as previously described using specific variables, including age, heart rate, systolic blood pressure, creatinine cardiac arrest at admission, ST segment deviation on ECG and elevated cardiac troponins^14^. AMI severity was determined using the Killip classification: Killip class I includes individuals with no clinical signs of heart failure; Killip class II includes individuals with rales or crackles in the lungs, an S3, and elevated jugular venous pressure; Killip class III describes individuals with frank acute pulmonary edema; and Killip class IV describes individuals in cardiogenic shock or hypotension (measured as a systolic blood pressure lower than 90 mmHg) and evidence of peripheral vasoconstriction (oliguria, cyanosis or sweating)^15^. Additional adverse outcomes included acute respiratory failure (ARF) that required ventilation support, failure of weaning from a ventilator (defined as administering mechanical ventilation during discharge), acute kidney injury (AKI, defined as serum creatinine elevated >0.3 mg/dl or 50% from baseline)^16^, and upper gastrointestinal bleeding (UBIG, defined as melena with positive occult blood examination, bright-red blood discharged from the nasogastric tube, or endoscopic evidence of mucosal bleeding).

### Determination of serum glucose levels, HbA1c and glycemic gaps

The glucose level upon admission was determined upon admission to the ED. HbA1c assays were performed using a blood analyzer (Primus CLC 385; Primus Corporation, Kansas City, MO, USA) equipped with a high-performance liquid chromatography system. The equation AG = 28.7 × HbA1c–46.7 was used to convert HbA1c levels to the estimated long-term average glucose levels (eAG) for the preceding 3 months^9^. The glycemic gap represents changes in serum glucose levels during the index event and was calculated from the glucose level at ED admission minus the eAG.

### Statistical analysis

Continuous data are expressed as the mean ± standard deviation and were analyzed using the two-tailed Student *t*-test. Categorical data are expressed as frequencies (%) and were evaluated using the chi-square test or Fisher’s exact test. A one-way analysis of variance was used to assess the significance of various characteristics, laboratory data, and adverse outcomes. A post-hoc analysis was performed using the Bonferroni test. A receiver–operator characteristic curve (ROC) curve was plotted to analyze the discriminative power of the prediction tools, and the area under the ROC (AUROC) and the corresponding 95% confidence intervals (CI) were calculated. Univariate and multivariate Cox hazard regression analyses were performed to identify the risk factors associated with MACEs. Variables with a p < 0.05 in the univariate analysis were entered into the multivariate Cox hazard regression analysis. The correlation between glycemic gap and continuous variables, such as BNP and GRACE scores, was evaluated by the Pearson product-moment correlation. The correlation between the glycemic gap and ordinal variables, such as Killip classes, was evaluated by the Spearman’s rank-order correlation. The data were analyzed using Statistical Package for the Social Sciences version 17.0 statistical software (SPSS, Inc., Chicago, IL, USA), and differences with p values < 0.05 were considered statistically significant. The log-rank test was used to determine the statistical significance of survival curves. The net reclassification improvement (NRI) was used to assess the improvement in model performance after adding parameters (MATLAB, MathWorks, Natick, MA, USA)^17^.

## Results

### Patient study population and clinical outcomes

We initially identified 441 patients with AMI and type 2 diabetes. One-hundred and ten patients were excluded because of missing HbA1c levels within 1 month prior to or immediately after admission (n = 92), the absence of plasma glucose levels at initial ED presentation (n = 6), hypoglycemia (n = 8), treatment with steroids (n = 2), or severe anemia requiring a blood transfusion (n = 2). Thus, we enrolled 331 patients after the chart review. Two hundred and fifty-four (76.7%) patients had NSTEMI, whereas 77 (23.3%) patients had STEMI. The demographic data and AMI-related clinical features, including GRACE scores and Killip classification, of the enrolled patients are shown in Table 1. Of these patients, 43 (13.0%) died during hospitalization and 61 (18.4%) experienced MACEs. Compared with survivors, non-survivors had a statistically significant older age, higher glycemic gap, maximal blood glucose during the first 48 hours and BNP level, and longer hospital stay.

### Correlations among acute hyperglycemia, glycemic gaps, long-term blood glucose control and major adverse cardiac events

**Figure 1:**
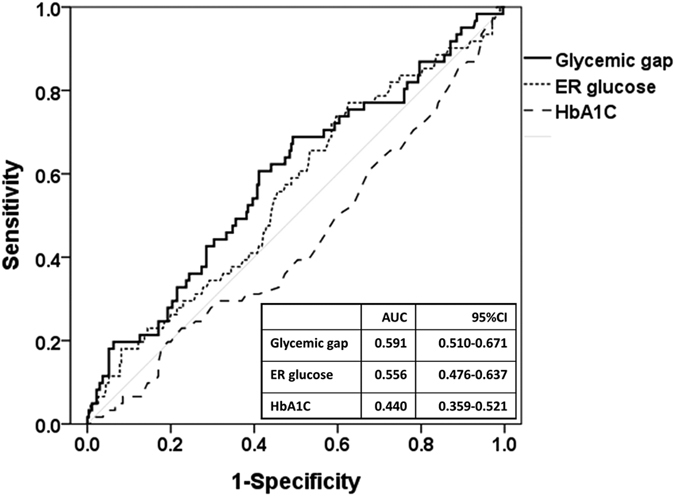
ROCs for acute hyperglycemia, chronic blood glucose control, glycemic gaps, and adverse outcomes in diabetic patients presenting to the emergency department with acute myocardial infarction. AUROC: area under the curve; ROC: receiver operating characteristic.

**Figure 2:**
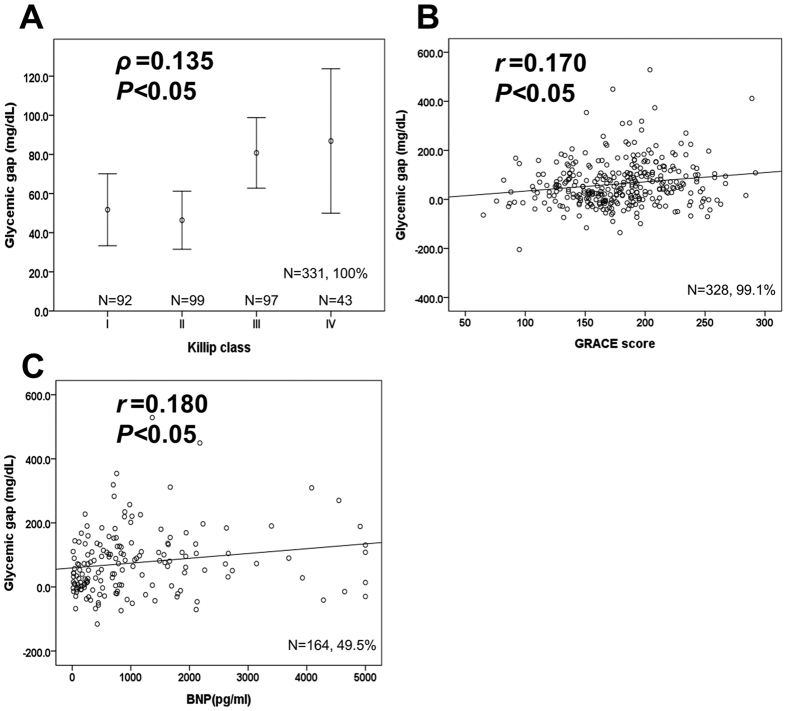
Correlations between glycemic gaps and Killip classification, GRACE scores and BNP levels.

**Figure 3:**
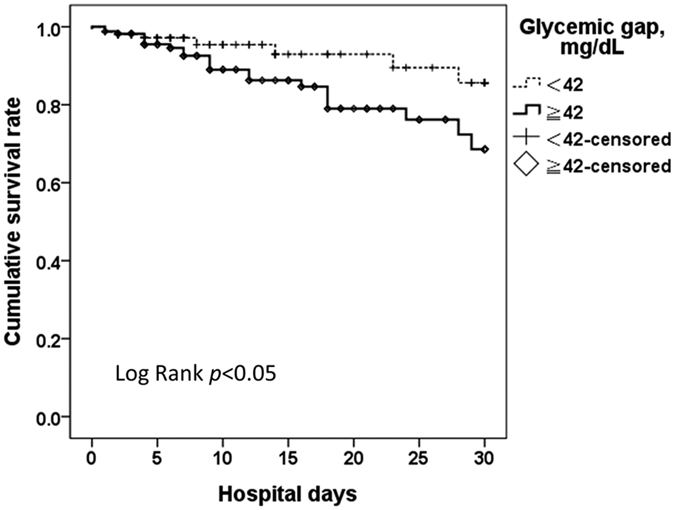
Kaplan–Meier survival curves of glycemic gaps in diabetic patients presenting to the emergency department with acute myocardial infarction.

As shown in Table 2, the Cox proportional hazard model revealed that the hazard ratio of the glycemic gap (mg/dL) for MACEs was 1.003 (95% CI: 1.000–1.005, p = 0.02). Compared with acute hyperglycemia (defined as blood glucose level of ≥250 mg/dL) and long-term glycemic controls, glycemic gaps showed greater AUROC values (0.591, 95%CI: 0.510–0.671) for MACEs occurrence (Fig. 1). We determined an optimal cutoff value of 42 mg/dL using the maximal Youden’s index with a sensitivity, specificity, positive predictive value and negative predictive value of 68.9%, 50.7%, 23.9% and 50.4%, respectively, for occurrence. There was no statistically significant difference in the comorbidity of patients with or without an elevated glycemic gap. As shown in Fig. 2, there were statistically significant correlations between the glycemic gap and GRACE score (r = 0.170, p < 0.05), Killip classification (r = 0.135, p < 0.05) and BNP (r = 0.180, p < 0.05). Only 164 patients (49.5%) had a BNP measurement because the levels of BNP were not routinely determined in ACS patients. As shown in Table 3, a further analysis revealed that an elevated glycemic gap (>42 mg/dL) was associated with males and cardiac arrest at ED admission. Patients with glycemic gaps >42 mg/dL showed a significantly higher incidence of in-hospital ARF (p = 0.003), mortality (p = 0.044), cardiogenic shock (p = 0.03), composed MACEs (p = 0.007), and GRACE scores (p < 0.001) and a lower LVEF (p = 0.04) when compared with patients with glycemic gaps <42 mg/dL. As shown in Fig. 3, a Kaplan–Meier survival curve showed that a glycemic gap > 42 mg/dL was associated with a significantly shorter survival when compared with a gap <42 mg/dL (log-rank test p < 0.05).

### Chronic blood glucose controls in diabetic patients with AMI

Unexpectedly, we found that chronic glycemic controls affected adverse outcomes and the length of ICU and hospital stays. Patients with good glycemic control, as determined by HbA1c values of <7%, had a statistically significant higher risk for AKI (p = 0.001), GRACE scores (p = 0.01) and hospital stays (p = 0.04) (Table 4). There were no statistically significant differences in glycemic gaps between these three HbA1c groups (p = 0.39). Admission hyperglycemia was statistically significantly associated with the development of MACEs only in patients with HbA1c ≤ 7% (HR: 1.003, 95% CI: 1.000–1.006, p=0.032), whereas there were no association between admission hyperglycemia and the development of MACEs in patients with 7% < HbA1c < 9% (HR: 1.002, 95% CI: 0.997–1.006, p = 0.47) and HbA1c ≥ 9% (HR: 1.004, 95% CI: 0.997–1.011, p = 0.22).

### Incorporating the glycemic gap into the TIMI and GRACE scores

**Figure 4:**
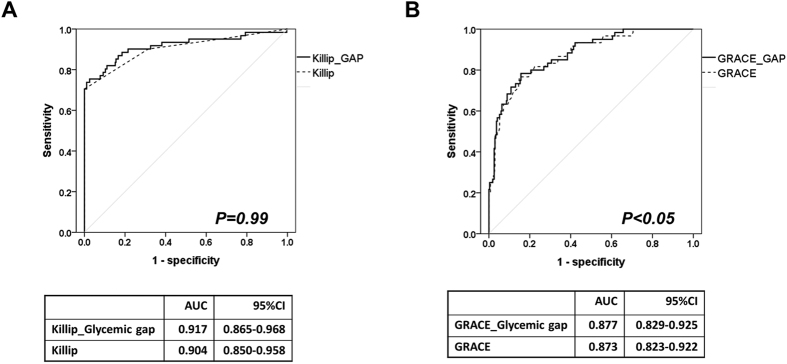
The effects of integrating glycemic gaps with the Killip classification and GRACE scores.

Consistent with previous studies, we found that GRACE scores and the Killip classification showed a superior discriminative performance at predicting MACEs in diabetic patients presenting to the ED with AMI. Incorporating the glycemic gap into the GRACE score significantly increased the AUROC from 0.873 (95% CI = 0.823−0.922) to 0.877 (95% CI = 0.829−0.925, NRI, 0.072, p: 0.028); however, the incorporation of the glycemic gap into the Killip classification did not result in a significant change in the AUROC: 0.904 (95% CI =  0.850−0.958) to 0.917 (95% CI = 0.865−0.968, p = 0.99) (Fig. 4).

## Discussion

There were three major findings in the present study, as follows: 1) the glycemic gap, by eliminating the possible influences of chronic blood glucose controls, supports the possible deteriorating effects of SIH on the early stage of AMI; 2) an elevated glycemic gap but not acute hyperglycemia was associated with increased mortality and the glycemic gap itself showed superior discriminative power for MACEs occurrence; and 3) the addition of the glycemic gap increased the discriminative power of the GRACE score in diabetic patients presenting to the ED with AMI.

Stress induced hyperglycemia can be attributed to the presence of excess levels of counter-regulatory hormones, anti-inflammatory cytokines, and increased gluconeogenesis and hepatic insulin resistance^5^,^18^,^19^. SIH could be a vicious cycle by increasing free fatty acids, insulin resistance, chemical inactivation of nitric oxide and the production of reactive oxygen species, a prothrombotic state, and vascular inflammation^20^. Patients with SIH could have increased susceptibility to myocardial ischemia-reperfusion injury due to increased oxidative stress, inflammation, and activation of stress-responsive kinases^21^. Acute hyperglycemia during coronary revascularization causes endothelial dysfunction and is associated with plaque instability and infarct size^22^. Even in non-diabetic patients with hip fracture, SIH was associated with increased risk of AMI^23^. Mechanistically, hyperglycemia decreases vascular dilation and increase permeability during the initial inflammatory responses, possibly through protein kinase C activation^24^. Glucose fluctuation can activate nuclear factor-kB and protein kinase C pathway, leading an increased expression of the adhesion molecules and excess formation of advanced glycation end products than stable glucose *in vitro*^25^,^26^. In addition, recent studies have shown that dermcidin, a stress induced protein, can inhibit glucose-induced insulin synthesis and result in hyperglycemia in patients with AMI and stress-induced type 1 diabetes^27^,^28^. Insulin can activate nitric oxide synthase and inhibit platelet aggregation and thus produce a beneficial effect on cardiovascular disease^29^. Aspirin can further increase plasma insulin levels by decreasing plasma dermcidin levels and reducing the infarct size in patients with AMI^27^,^30^. Our finding of that admission hyperglycemia was statistical significantly associated with the development of MACEs only in patients with HbA1c ≤ 7% further supported the hypothesis that SIH would worsen the prognosis. Recent clinical trials of insulin treatment in AMI patients have resulted in varying levels of benefit, but the clinical benefit of an aggressive insulin treatment remains unproven^4^,^20^,^31^. We thought that reevaluation of those trials by using glycemic gap might also help in elucidating the potential benefits in acute control of blood glucose. In non-diabetic or mixed populations, elevated admission glucose levels are common in patients with AMI and are strongly associated with an increased risk of poorer outcomes and mortality^2^,^32^. Acute and mean hyperglycemia during hospitalization is associated with adverse clinical outcomes^2^,^33^,^34^,^35^. Elevated admission hyperglycemia was associated with in-hospital mortality, a lower myocardial salvage index and composite MACEs in acute ST-segment elevation myocardial infarction (STEMI) patients^36^,^37^,^38^,^39^,^40^. Patients with a Killip class of III or IV had statistically significant higher first blood glucose levels and higher in-hospital mortality when compared with Killip class I patients^15^. Nonetheless, several studies revealed that acute hyperglycemia may not reflect the severity of AMI in patients with diabetes^36^,^41^. A prospective multicenter Korean study found that admission hyperglycemia predicted the 30-day mortality in 816 non-diabetic STEMI patients with cardiogenic shock but not diabetic patients^42^. There was no significant difference in myocardial salvage after PCI between diabetic patients with or without SIH^36^. Intensive glucose regulation did not reduce the infarct size in hyperglycemic patients with AMI treated with PCI but rather was associated with harm^43^. We believe that one explanation for these discordant study results could be lack of consideration of the chronic blood glucose levels in patients with diabetes. By using the glycemic gap, we eliminated the possible influence of chronic hyperglycemia in diabetic patients. The glycemic gap may explain the “diabetes paradox” and the association between acute hyperglycemia, long-term glucose controls, and certain adverse clinical outcomes. Once a novel biomarker becomes available to facilitate risk prediction, it is essential to compare this marker with existing tools, i.e., combining different biomarkers and clinical scores to further increase the AUROC^44^,^45^. In the NRI analysis, incorporation of the glycemic gap into the GRACE score could increase the AUROC for the development of MACEs, suggesting that the glycemic gap may be used as an additional prognostic marker in diabetic patients under physiological stress.

The “diabetes paradox,” i.e., diabetes and glycemic control are not independently associated with mortality in critical ill patients, was proposed by Krinsly *et al*. and has been continuously observed in other settings^8^,^46^,^47^. The HbA1c level is characterized by lower biological variability and is relatively unaffected by acute stress or sepsis^12^. Notably, HbA1c values can be affected by anemia and blood transfusion^48^,^49^. In patients without diabetes, elevated glucose at admission or acute hyperglycemia was associated with adverse outcomes in patients with acute critical illnesses. Acute hyperglycemia is linked to increased adverse outcomes in patients with trauma^42^,^50^, poorer neurological improvement and symptomatic hemorrhage in patients with acute ischemic stroke^51^, and increased risk of nosocomial complications in patients with community acquired pneumonia (CAP)^10^,^52^. Admission basal glucose levels were independently associated with increased mortality in ED patients without a diagnosis of diabetes^53^. Nonetheless, several studies have reported that the relationship between hyperglycemia and acute illness-related mortality is relatively weak in diabetic critically ill patients^54^,^55^,^56^,^57^,^58^. There was no independent association between hyperglycemia and mortality once lactate levels were controlled^58^. Egi *et al*. reported that ICU mortality was not associated with diabetes *per se* or chronic blood glucose control. Freire *et al*. demonstrated that the highest glucose value during the first 24 h after ICU admission did not predict hospital mortality in the medical ICU^59^. Patients with chronic obstructive lung diseases treated with corticosteroids developed significant hyperglycemia, but the increase in blood glucose levels did not correlate with the maximum dose of corticosteroids and were not associated with mortality, length of hospital stay, or re-admission rates^60^. The duration of hyperglycemia or the amount of insulin given did not have a major impact on the outcome of patients with primary acute neuromuscular respiratory failure^61^. Consistent with our previous work that showed elevated glycemic gaps were associated with adverse outcomes in diabetic patients with liver abscesses, CAP and ICU stays^62^,^63^,^64^, we found that elevated glycemic gaps were also associated with MACEs in diabetic patients with AMI. We speculate that the acute surge of glucose levels beyond the long-term average (or in nondiabetic patients) should be used as a surrogate marker for acute physiological stress. Furthermore, pre-existing hyperglycemia in diabetic patients may be a confounding factor for the prediction of MACEs in patients with AMI.

Chronic hyperglycemia had been shown to associate with smaller peak creatine kinase levels in patients with acute hyperglycemia^65^. Consistent with previous studies regarding chronic hyperglycemia assessed by HbA1c could not predict infarct size, short-term outcomes or in-hospital mortality in patients with AMI^65^,^66^,^67^,^68^, we again revealed that improved blood glucose control was not associated with superior outcomes in patients with AMI. While HbA1c has strong association with long-term prognosis, the short-term prognostic effects might have been weakened by the relative shorter study period in a limited number of patients^69^,^70^. An elevated HbA1c level had also been shown to not associated with a higher incidence of contrast induced-acute kidney injury compared to optimal HbA1c levels in patients with type 2 diabetes^71^. The possible adverse consequences of more stringent glycemic controls in diabetic patients have been further illustrated in two studies. In the Action in Diabetes and Vascular Diseases: Preterax and Diamicron Modified Release Controlled Evaluation (ADVANCE) and Action to Control Cardiovascular Risk in Diabetes (ACCORD) studies, aggressive glucose control to reduce the HbA1c levels from 7.5 to 6.5% and 8.3 to 6.4% had resulted in only nonsignificant beneficial trend in mortality and even significantly increased mortality respectively^72^,^73^. Several hypothetical reasons have been proposed to explain the lack of an association between HbA1c levels and outcomes in previously mentioned and current studies. Physiological and cellular readjustments in response to hyperglycemia occur over time in diabetic patients and the speed of correction of hyperglycemia in readjusted diabetic patients with higher HbA1c might adversely affect the outcomes^74^. We speculate that the readjustment of the blood glucose to a higher set-point value might influence the subsequent biological and clinical effects of acute fluctuation of blood glucose, possibly exert paradoxical protective effects during an acute stress, and hence result in lower GRACE scores and shorter hospital stays. The potential adverse influence of uncontrolled DM on platelet reactivity might have been mitigated by dual antiplatelet therapy used universally in the reperfusion era^75^,^76^,^77^. Mechanistically, decreased activity of the sodium proton exchanger in diabetic myocardium might attenuate reperfusion injury and therefore paradoxically prevents the diabetic heart from ischemic insults^78^. Since there were no statistically significant differences in the levels of glycemic gaps between HbA1c groups, higher MACEs in patients with better chronic blood glucose control cannot be attributed to lower ADAG levels alone.

### Limitations

Our study has several limitations. First, it was a retrospective design and thus may have been subjected to selection bias. We believe that a prospective study of glycemic gaps in both diabetic and non-diabetes patients should be conducted to clarify the usefulness of the glycemic gap as a biomarker. Second, the adequacy of glycemic control during hospitalization might have influenced the outcomes. In the present study, we did not specifically address the effect of glycemic control during hospitalization. Although the diagnostic performance of glycemic gaps in this AMI setting was unfavorable when compared with our previous studies^62^,^63^,^64^, we demonstrated that glycemic gaps were superior to acute hyperglycemia as a risk predictor in diabetic patients. We acknowledge that the ADAG study was originally designed to investigate the correlation between HbA1c levels and average capillary glucose levels^9^. The capillary glucose level is slightly higher than serum glucose levels in critically ill ICU patients. The correlation coefficient between capillary and serum glucose levels was 0.911^79^. Another study revealed that the association between venous and capillary glucose values varied. In people with type 2 diabetes, venous plasma was higher than capillary blood for random and fasting samples and lower for samples collected 2 hours after oral glucose^80^. The discrepancy between serum blood glucose levels and capillary whole blood glucose levels was less than 5% during a semiannual quality control project conducted at our institution. Although the correlation between capillary and serum glucose levels was good, the data should be interpreted with caution. The serum levels of insulin were not routinely determined in patients with AMI; thus, we were unable to elucidate the association between glycemic gaps and hyperinsulinemia. We believe that incorporating serum insulin levels and insulin resistance studies would provide insight into the pathophysiology of SIH. Notably, our study population had higher levels of creatinine and BNP when compared with other AMI studies. One explanation for this finding is that there were 50.1% of the patients in the present study had chronic kidney disease (CKD). The prevalence of CKD was compatible with previous studies regarding diabetic patients presenting with AMI (39.9 to 77%)^81^,^82^,^83^,^84^. The plasma concentration of BNP is commonly elevated in patients with CKD and correlates weakly with renal function^85^. Nonetheless, the plasma BNP level is a reliable marker of left ventricle overload in patients with CKD^86^. The selection bias of BNP measurements might have resulted in higher values in our study, i.e., those patients who had plasma BNP determinations in the ER (49.5%) might have had more severe heart failure.

## Conclusion

Elevated glycemic gaps between admission serum glucose levels and the ADAG were associated with an increased risk of mortality in patients with diabetes presenting with AMI. We suggest that the glycemic gap could be further studied as an adjunct assessment to determine the severity and prognosis of diabetic patients presenting with AMI. The association between glycemic gap, chronic blood glucose controls and the outcomes should be further explored in prospective longitudinal studies.

## Additional Information

**How to cite this article**: Liao, W.-I. *et al*. An Elevated Glycemic Gap is Associated with Adverse Outcomes in Diabetic Patients with Acute Myocardial Infarction. *Sci. Rep.* **6**, 27770; doi: 10.1038/srep27770 (2016).

## Figures and Tables

**Table 1:** Comparison of the characteristics of survivors and non-survivors.

	Survivors (n = 288)	Non-survivors (n = 43)	*p* value
Age (yrs)	68.8 ± 12.7	77.7 ± 13.1	<0.001*
Male	193(67.0%)	24(55.8%)	0.15
Hypertension	251(87.2%)	39(90.7%)	0.51
Smoking	141(49.0%)	15(34.9%)	0.09
Dyslipidemia	160(55.6%)	13(30.2%)	0.002*
Family history of CAD	18(6.3%)	0(0%)	0.09
Glycemic gap, mg/dL	58.3 ± 84.8	95.7 ± 119.8	0.01*
Admission glucose, mg/dL	227.6 ± 97.8	260.7 ± 131.5	0.12
Max. glucose during first 48 h, mg/dL	281.5 ± 98.9	328.1 ± 118.8	0.005*
HbA1c, %	7.5 ± 1.7	7.4 ± 1.7	0.59
BNP, pg/ml	940 ± 1045	1967 ± 1571	0.005*
Hb, g/dL	12.5 ± 2.7	11.4 ± 2.7	0.01*
Cr, mg/dL	2.8 ± 3.7	2.9 ± 2.8	0.94
Alb, g/dL	3.3 ± 0.46	3.1 ± 0.59	0.10
Total cholesterol, mg/dL	165.6 ± 56.5	136.9 ± 39.5	0.006*
Triglycerol, mg/dL	163.6 ± 202.9	99.6 ± 41.1	0.08
Bilirubin, mg/dL	0.77 ± 0.8	1.11 ± 1.4	0.17
LDL, mg/dL	104.1 ± 40.5	89.6 ± 43.6	0.13
HDL, mg/dL	37.7 ± 11.6	40.1 ± 10.7	0.56
Uric acid, mg/dL	6.5 ± 3.5	7.1 ± 2.4	0.39
Peak CK, U/L	1109 ± 2073	809 ± 1133	0.36
Peak troponin-I, ng/mL	24.1 ± 33.0	28.3 ± 37.2	0.45
STEMI	71(24.7%)	6(14.0%)	0.17
Killip class			<0.001*
I	92(31.9%)	0(0%)	
II	95(33.0%)	4(9.3%)	
III	85(29.5%)	12(27.9%)	
IV	16(5.6%)	27(62.8%)	
Number of Diseased vessels			0.24
Single-vessel	26(10.9%)	2(6.9%)	
Double-vessel	61(25.5%)	4(13.8%)	
Triple-vessel	152(63.6%)	23(79.3%)	
GRACE scores	171.4 ± 1.2	224.1 ± 1.2	<0.001*
Hospital stay (days)	13.2 ± 17.8	25.0 ± 27.3	0.008*

^*^P < 0.05

CAD, coronary artery disease; Max., maximum; BNP, brain natriuretic peptide; LDL, low density lipoprotein cholesterol; HDL, high density lipoprotein cholesterol; CK, creatine kinase; STEMI, ST segment elevation myocardial infarction; GRACE, global registry of acute coronary events.

**Table 2:** Univariate and multivariate hazard regression analyses for the development of major adverse cardiovascular events.

	Univariate	*p-*value		Multivariate	*p-*value
	HR(95% CI)			HR(95% CI)	
Age	1.012(0.991–1.034)	0.26	Age	0.986(0.965–1.009)	0.24
Gender	0.983(0.582–1.660)	0.95	Gender	0.898(0.510–1.579)	0.71
Hypertension	1.872(0.829–4.226)	0.13	Glycemic gaps	1.002(1.000–1.005)	0.036 *
Smoking	1.189(0.705–2.007)	0.52	Peak Troponin–I	1.001(0.994–1.008)	0.81
Dyslipidemia	1.462(0.848–2.512)	0.17	Grace scores	1.005(0.994–1.016)	0.40
Family history of CAD	2.383(0.329–17.264)	0.39	Killip class	5.022(2.874–8.775)	<0.001*
Glycemic gaps	1.003(1.000–1.005)	0.02*			
Admission glucose	1.002(1.000–1.004)	0.056			
HbA1c	0.983(0.833–1.160)	0.83			
Hb	1.049(0.949–1.160)	0.35			
Total cholesterol	0.994(0.986–1.001)	0.08			
Peak troponin–I	1.008(1.001–1.014)	0.03*			
BNP	1.000(1.000–1.000)	0.55			
STEMI	0.597(0.333–1.071)	0.08			
Grace scores	1.027(1.019–1.034)	<0.001*			
Killip class	5.391(3.545–8.198)	<0.001*			

CI, confidence interval; HR, Hazard ratio; CAD, coronary artery disease; BNP, brain natriuretic peptide; STEMI, ST segment elevation myocardial infarction; GRACE, global registry of acute coronary events. *p < 0.05.

**Table 3:** Characteristics and clinical outcomes versus glycemic gaps in patients with both diabetes and acute myocardial infarction.

	Glucose–ADAG < 42 mg/dL (n = 155)	Glucose–ADAG ≥ 42 mg/dL (n = 176)	*p* value
Age (yrs)	68.9 ± 13.6	70.9 ± 12.7	0.16
Male	111(71.6%)	106(60.2%)	0.03*
Hypertension	135(87.1%)	155(88.1%)	0.79
Smoking	82(52.9%)	74(42.0%)	0.051
Dyslipidemia	84(54.2%)	89(50.6%)	0.51
Family history of CAD	9(5.8%)	9(5.1%)	0.78
BNP	877± 1203	1245 ± 1159	0.050
LVEF, %	46.6 ± 14.8	42.8 ± 14.8	0.04*
GRACE scores	171.4 ± 1.2	224.1 ± 1.2	<0.001*
Killip class			0.006*
I	49(31.6%)	43(24.4%)	
II	56(36.1%)	43(24.4%)	
III	34(21.9%)	63(35.8%)	
IV	16(10.3%)	27(15.3%)	
PCI	127(81.9%)	141(80.1%)	0.67
Number of Diseased vessels			0.82
Single-vessel	13(10.2%)	15(10.6%)	
Double-vessel	33(26.0%)	32(22.7%)	
Triple-vessel	81(63.8%)	94(66.7%)	
MACEs	19(12.3%)	42(23.9%)	0.007*
Mortality	14(9.0%)	29(16.5%)	0.044*
Cardiogenic shock	17(11.0%)	35(19.9%)	0.03*
Cardiac arrest at admission	0(0%)	6(3.4%)	0.02*
VF, AV block, resuscitation	40(25.8%)	54(30.7%)	0.33
Acute heart failure	106(68.4%)	133(75.6%)	0.18
Acute respiratory failure	28(18.1%)	57(32.4%)	0.003*
UGIB	13(8.4%)	20(11.4%)	0.38
Acute kidney injury	81(45.8%)	96(54.2%)	0.68
Hospital stay (days)	13.3 ± 18.8	16.0 ± 20.5	0.21

^*^P < 0.05

AMI, acute myocardial infarction; CAD, coronary artery disease; MACEs, major adverse cardiac events; UGIB, upper gastrointestinal bleeding; PCI, percutaneous coronary intervention; BNP, brain natriuretic peptide; GRACE, global registry of acute coronary events; LVEF, left ventricular ejection fraction.

**Table 4:** Clinical outcomes versus chronic glycemic control in patients with both diabetes and acute myocardial infarction.

	>HbA1c ≤ 7% (n = 156)	7% < HbA1c < 9% (n = 122)	HbA1c ≥ 9% (n = 53)	*p* value
Mortality	20(12.8%)	18(14.8%)	5(9.4%)	0.63
Cardiac arrest at admission	4(2.6%)	1(0.8%)	1(1.9%)	0.55
Cardiogenic shock	32(20.5%)	13(10.7%)	7(13.2%)	0.07
MACEs	35(22.4%)	19(15.6%)	7(13.2%)	0.19
VF, AV block, resuscitation	51(32.7%)	30(24.6%)	13(24.5%)	0.26
Acute respiratory failure	46(29.5%)	27(22.1%)	12(22.6%)	0.33
Acute heart failure	122(78.2%)	81(66.4%)	36(67.9%)	0.07
UGIB	17(10.9%)	14(11.5%)	2(3.8%)	0.26
Acute kidney injury	92(59.0%)	69(56.6%)	16(30.2%)	0.001*
PCI	125(80.1%)	103(84.4%)	40(75.5%)	0.36
GRACE score	183 ± 38	176 ± 41	164 ± 43	0.01*
Hospital stay (days)	17.0 ± 21.3	14.2 ± 20.3	9.0 ± 10.6	0.04*
Glycemic gap (mg/dL)	69.1 ± 93.8	54.2 ± 79.7	66.3 ± 105.1	0.39

^*^P < 0.05

MACEs, major adverse cardiac events; UGIB, upper gastrointestinal bleeding; PCI, percutaneous coronary intervention; GRACE, global registry of acute coronary events.
